# Standardising the measurement of physical activity in people receiving haemodialysis: considerations for research and practice

**DOI:** 10.1186/s12882-019-1634-1

**Published:** 2019-12-04

**Authors:** Hannah M. L. Young, Mark W. Orme, Yan Song, Maurice Dungey, James O. Burton, Alice C. Smith, Sally J. Singh

**Affiliations:** 1 Department of Respiratory Science, University of Leicester, Leicester Kidney Lifestyle Team, Academic Unit, Leicester General Hospital, Gwendolen Road, Leicester, LE4 5PW UK; 2 Centre for Exercise and Rehabilitation Science, NIHR Leicester Biomedical Research Centre - Respiratory, Glenfield Hospital, University Hospitals of Leicester NHS Trust, Leicester, UK; 3 Department of Health Sciences, University of Leicester, Leicester, UK; 4 Nantong University, Nantong, China; 5 Department of Cardiovascular Science, University of Leicester, Leicester, UK; 6 National Centre for Sport and Exercise Medicine, Loughborough University, Loughborough, UK

**Keywords:** Accelerometry, End-stage renal disease, Exercise, Haemodialysis, Physical activity

## Abstract

**Background:**

Physical activity (PA) is exceptionally low amongst the haemodialysis (HD) population, and physical inactivity is a powerful predictor of mortality, making it a prime focus for intervention. Objective measurement of PA using accelerometers is increasing, but standard reporting guidelines essential to effectively evaluate, compare and synthesise the effects of PA interventions are lacking. This study aims to (i) determine the measurement and processing guidance required to ensure representative PA data amongst a diverse HD population, and; (ii) to assess adherence to PA monitor wear amongst HD patients.

**Methods:**

Clinically stable HD patients from the UK and China wore a SenseWear Armband accelerometer for 7 days. Step count between days (HD, Weekday, Weekend) were compared using repeated measures ANCOVA. Intraclass correlation coefficients (ICCs) determined reliability (≥0.80 acceptable). Spearman-Brown prophecy formula, in conjunction with a priori ≥  80% sample size retention, identified the minimum number of days required for representative PA data.

**Results:**

Seventy-seven patients (64% men, mean ± SD age 56 ± 14 years, median (interquartile range) time on HD 40 (19–72) months, 40% Chinese, 60% British) participated. Participants took fewer steps on HD days compared with non-HD weekdays and weekend days (3402 [95% CI 2665–4140], 4914 [95% CI 3940–5887], 4633 [95% CI 3558–5707] steps/day, respectively, *p* < 0.001). PA on HD days were less variable than non-HD days, (ICC 0.723–0.839 versus 0.559–0.611) with ≥ 1 HD day and ≥  3 non-HD days required to provide representative data. Using these criteria, the most stringent wear-time retaining ≥ 80% of the sample was ≥7 h.

**Conclusions:**

At group level, a wear-time of ≥7 h on ≥1HD day and ≥ 3 non-HD days is required to provide reliable PA data whilst retaining an acceptable sample size. PA is low across both HD and non- HD days and future research should focus on interventions designed to increase physical activity in both the intra and interdialytic period.

## Background

Physical activity (PA), defined as “any bodily movement produced by skeletal muscle which results in caloric expenditure” [1], is exceptionally low amongst the haemodialysis (HD) population and exacerbated by enforced inactivity during HD treatment [2, 3]. Cardiovascular disease is the leading cause of death in people receiving HD, which may be compounded by a physically inactive lifestyle [2, 4]. Indeed, physical inactivity is a powerful predictor of mortality in people receiving HD [4, 5] and is further associated with other outcomes, including reduced quality of life [6, 7], increased risk of hospitalisation [4, 5] and reduced muscle mass [8], making it a prime focus for intervention. Recent guidance recommends all people living with chronic kidney disease, including those receiving HD, be encouraged to participate in regular PA and exercise [9–11].

Given its importance, a valid [12], unobtrusive and feasible method of measuring PA [13] is required. Consequently, accelerometers are gaining popularity for the quantification of human movement. Several studies have objectively measured PA in the HD population, identifying that PA is significantly lower in this group than in matched sedentary controls [3, 14–19], and is lowest on HD days [3, 16, 18–21]. However, there is currently no guidance on the reporting and processing of data, which is inclusive of a geographically and ethnically diverse HD population. Minimum wear time and number of valid days criteria have been recommended in other chronic diseases [22, 23], but these may not be appropriate for use in the HD population, where PA is restricted by HD treatment. PA behaviour is inherently variable and therefore understanding the number of days and hours of measurement required to obtain representative data is critical to enhancing data quality. This will establish a better understanding of factors associated with PA to help target and tailor interventions, and promote the effective evaluation, comparison and synthesis of PA interventions. Valid wear times are universally inconsistent and scarcely reported in the HD literature, ranging from 8 to 24 hours [3, 16, 19, 24]. The minimum number of valid days also varies considerably; between 0 and 3 HD days and 1–4 non-HD days [3, 16, 19, 21, 25, 26]. Adherence with wearing the monitor, which inherently impacts the amount of PA data it is possible to collect, is also rarely reported within HD studies. The collection of PA data in HD patients is challenging due to low adherence [27], but the impact of poor concordance with the monitor, and how to address this in the measurement and analyses of PA data, is unknown and requires further investigation.

Additionally, most studies that have used objective PA measurement have focused primarily upon middle-to-high income Western countries [3, 14–19, 21, 24], despite a growing prevalence of end-stage renal disease, an exponential increase in the demand for HD [28, 29] and high levels of self-reported physical inactivity within East Asia [26]. It is widely recognised in global surveillance PA data from healthy populations that demographic, geographical and cultural variation in PA levels exists. To date, a single observational study from Japan found no difference between PA levels on HD days and non-HD days, which differs from patterns of PA in Western HD populations [8], highlighting the need for PA measurement guidance in the HD population that reflects this diversity [30].

The aims of this study were to (i) determine the measurement and processing guidance to ensure representative PA data amongst a diverse population of people receiving HD, accounting for the potential differences between HD days, weekdays and weekend days and; (ii) to assess adherence to PA monitor wear amongst HD patients.

## Methods

### Research design

Data were collected between 2013 and 2016 and formed a convenience sample comprised of data from participants enrolled in a cross-sectional observational study of PA in a multi-ethnic UK and Chinese population receiving HD (International Standard Randomised Controlled Trial Number:11615440), and baseline data from a previously reported trial of intradialytic exercise [31]. Therefore, no formal sample size calculation was performed for this study. Both studies were approved by the NHS Research Ethics Committee (South East Scotland; 14/EM/1049 and Northampton; 11/EM/0149) and the Ethical Committee of the Affiliated Hospital of Nantong University (Ref 2015–12). All participants provided written informed consent.

### Participants and settings

Participants were recruited from HD units in Leicester, UK and Nantong, China. All participants were prevalent patients aged over 18 years of age. HD was performed thrice weekly for four hours in all participants. Participants were excluded if: they were unable to provide informed consent or, to wear the accelerometer; if they presented with established contraindications to exercise [32]; clinically overt infection within the last six weeks; or had lower limb vascular access.

### Recruitment

Eligible participants were identified by their supervising Nephrologist. The study team provided eligible participants with the study information sheet and, at least 48 h later, invited them to take part.

### Physical activity

PA was measured using the SenseWear Armband (SWA) Pro 3 (BodyMedia, Inc., Pittsburgh PA, USA). The SWA is a validated multisensor monitor which combines information from a biaxial accelerometer with other sensors measuring heat flux, temperature and galvanic skin response, and widely used in HD groups [12, 33]. Participants were instructed to wear the armband as per manufacturer instructions on their vascular access-free arm. They were instructed to wear the monitor for 7 consecutive days, which included their usual HD treatment sessions. Participants were asked to continue to follow their usual care schedules, removing the armband for bathing only.

Data from the SWA were processed in 60-s epochs. Non-wear was identified automatically by the SWA from a loss of physiological parameters. Step count and waking wear time (defined as total wear time minus sleep according to SWA proprietary algorithm) were extracted for each day the device was worn for at least one hour.

Average step counts were created for all days for each participant across a range of minimum wear time criteria; ≥1 h to ≥12 h in one-hour increments. Variability in PA across different days was examined by classifying days as either HD days (days on which participants received HD treatment), weekdays (Monday to Fridays when HD treatment not received), and weekends (Sunday for all patients and additionally Saturday for those who received HD treatment in the Monday, Wednesday, Friday cohort). Differences PA between these types of days, the variability in wear time and step count was examined using hourly summaries. Where HD session times were recorded, adherence to the monitor during HD sessions was assessed**.**

### Other measures

Patient demographic and clinical data were extracted from participants’ medical records. Co-morbidity was assessed using the Charlson Comorbidity Index (CCI) modified for use in people receiving HD [34].

### Data processing and analysis

Sample characteristics are presented as mean ± standard deviation, median (IQR) or n (%), as appropriate. The first step of determining measurement guidance was to determine differences between types of day across a range of minimum wear time criteria for step count and wear time, and differences in wear-time and step count between wear time criteria for each type of day, in order to understand how PA data should be organised for analysis. These analyses were conducted using repeated-measures analysis of covariance (ANCOVA; waking wear time). Greenhouse-Geisser adjusted F was used to determine statistical significance and post-hoc Bonferroni pairwise comparisons used to identify pairwise differences.

Following this, intraclass correlation coefficients (ICC; two-way mixed; consistency) were calculated across a range of wear time criteria for each type of day. The minimum number of days required to obtain representative PA data (represented by an ICC ≥0.80) [35] was estimated using the Spearman-Brown prophecy formula [36, 37]. The influence of increasing wear time and number of valid days on sample size retention was also examined, with an acceptable sample size retention set at 80%. All statistical analyses were performed using SPSS 24 (IBM UK Ltd., UK) with alpha set at 0.05.

## Results

### Recruitment and participant flow

**Fig. 1:**
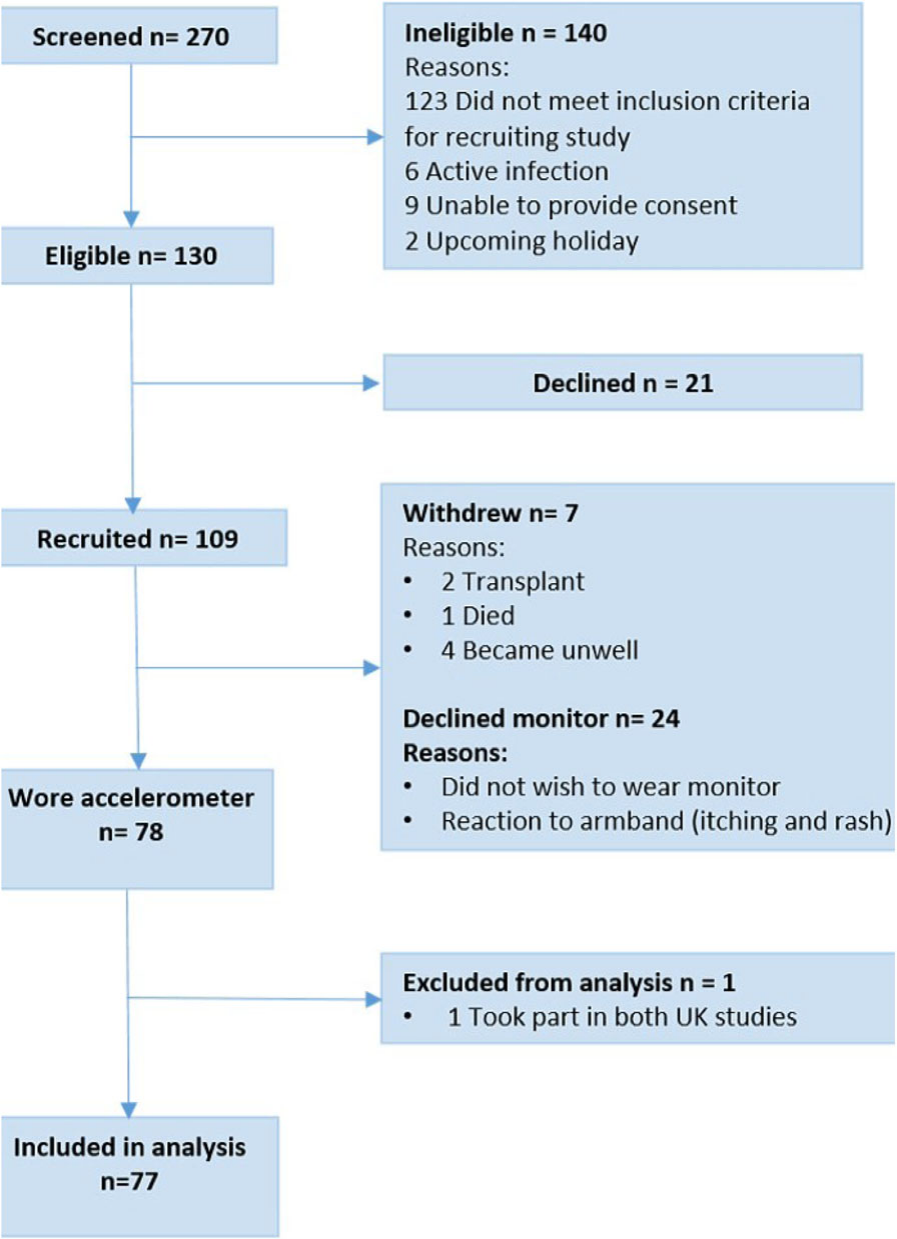
CONSORT flow diagram

PA data was available for 77 participants (Fig. 1). Participants wore the activity monitor each day for an average 13.8 ± 0.38 waking hours and took an average of 4010 ± 3145 steps/day.

### Participant characteristics

Participants demographic characteristics are outlined in Table 1. The majority (*N* = 49, 64%) of participants were male and the mean age was 56 ± 14. UK-based participants were from ethnically diverse backgrounds, comprising participants who were primarily of White British (*N* = 26, 56% of the UK sample) and Indian descent (*N* = 17, 37% of the UK sample). The most frequent primary causes of Chronic Kidney Disease were Glomerulonephritis (*N* = 31, 40%) and Diabetic Nephropathy (*N* = 11, 14%). Participants had been receiving HD for a median of 40 months (19–72). They had a low burden of co-morbid disease (CCI score 3, interquartile range 2–5), the most commonly reported co-morbidities were hypertension (*N* = 53, 32%), diabetes (*N* = 16, 10%) and ischaemic heart disease (*N* = 13, 8%).

**Table 1:** Participant characteristics

	Total (*n* = 77)
Age (years)^a^	56 ± 14
Sex n (%)	
Female	28 (36)
Male	49 (64)
Country n (%)	
British	
White British	26 (34)
Indian	17 (22)
Any other Asian background	1 (1)
Caribbean	1 (1)
Any other black background	1 (1)
Chinese	31 (40)
Diagnosis n (%)	
Glomerulonephritis	31 (40)
Diabetic Nephropathy	11 (14)
Renal vascular disease	9 (12)
Aetiology unknown	8 (10)
Polycystic Kidney Disease	7 (9)
Chronic Pyelonephritis	5 (7)
Membranous nephropathy	2 (3)
Light chain deposition disease	1 (1)
Systemic Lupus Erythematosus	1 (1)
Cyclosporine A Nephropathy	1 (1)
Other genetic kidney disease	1 (1)
Time on HD (months)	40 (19–72)
BMI (kg/m^2^)	24.00 (21.30–28.40)
Charlson comorbidity index	3 (2–5)
Previous transplant n (%)	
No	68 (88)
Yes	9 (12)
Haemoglobin^a^ (g/dl)	11.57 ± 1.48
Albumin^a^ (g/l)	39.19 ± 4.49
CRP (mg/L)	5.00 (3.75–7.00)
Use of ESA n (%)	
Yes	69 (90)
No	8 (10)

Data reported as median (interquartile range) unless stated. ^a^mean and SD. Abbreviations: *BMI* Body mass index, *CRP* C-Reactive Protein, *ESA* Erythropoietin stimulating agent, *HD* haemodialysis

### Comparison of wear time between haemodialysis days, weekdays and weekends

Across all wear time criteria, participants wore the monitor for significantly fewer minutes on HD days (803 [95% CI 759–847] minutes/day) than weekdays (951 [95% CI 913–989] minutes/day, *p* < 0.001) and weekends (972 [95% CI 934–1009] minutes/day *p* < 0.001) (Additional file 1: Table S1). During the four-hour HD sessions for (*N* = 60, 78% of the sample), the SWA was not worn for 1.5 ± 1.3 h/HD session (37% of an HD session).

### Comparison of step count between haemodialysis days weekdays and weekends

**Fig. 2:**
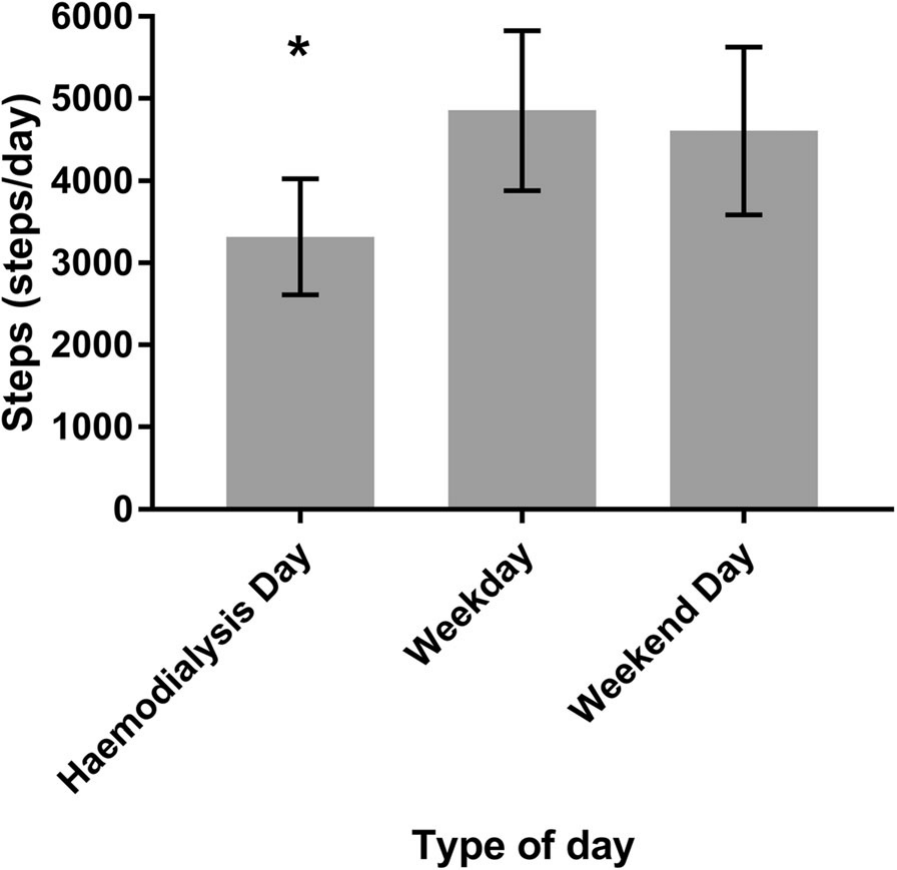
Adjusted average daily step count. Data presented as mean (95%CI) for haemodialysis days (days on which participants received haemodialysis treatment), weekdays (Monday to Fridays when HD treatment not received) and weekend days (Sunday for all patients and additionally Saturday for those who received HD treatment in the Monday, Wednesday, Friday cohort)

Step count on HD days (3402 [95%CI 2665–4140] steps/day) was significantly lower than on weekdays across all wear time criteria (4914 [95%CI 3940–5887] steps/day, *p* < 0.001) and weekend days (4633 [95%CI 3558–5707] steps/day, *p* < 0.001) (Fig. 2, Additional file 2: Table S2). Findings were unchanged after controlling for waking wear time (Additional file 3: Table S3). Weekday and weekend wear time and step count data were subsequently pooled to form non-HD days, used from this point forward.

### Variance in step count between types of day

Based on all three HD days, all ICCs from a wear time criteria ≥ 7 h were > 0.80 (≥ 7 h ICC 0.815), Table 2), indicating that one HD day was sufficient to provide representative PA data (Table 2). For all four non-HD days, ICCs were lower (eg ≥ 7 h ICC 0.591, Table 2), indicating that a single day of data is insufficient. Using the Spearman-Brown formula, at least 3 non-HD days (out of a possible four) were required to obtain representative PA data.

**Table 2:** Changes in step count per HD and non-HD day, intraclass correlation coefficients and number of days of activity monitoring required to obtain representative PA data across a range of minimum wear time criteria

	HD step count				ICCs	Number of days needed^a^	Number of days needed^b^	Non-HD step count				ICCs	Number of days needed^a^	Number of days needed^b^
Wear time criteria (hours)	N	Day 1	Day 2	Day 3				N	Day 1	Day 2	Day 3			
≥ 1	62	3249 ± 3481	3896 ± 3266	3050 ± 3195	0.723	1.53	2	65	5126 ± 5173	3499 ± 3992	4906 ± 4688	0.559	3.16	4
≥ 2	60	3318 ± 3518	3993 ± 3272	3139 ± 3210	0.719	1.56	2	64	5023 ± 4977	3462 ± 4026	4713 ± 4458	0.586	2.83	3
≥ 3	57	3308 ± 3610	3986 ± 3355	3207 ± 3270	0.723	1.53	2	63	5024 ± 5017	3517 ± 4034	4714 ± 4494	0.588	2.80	3
≥ 4	55	3288 ± 3670	3912 ± 3350	3241 ± 3299	0.744	1.38	2	63	5024 ± 5017	3517 ± 4034	4714 ± 4494	0.588	2.80	3
≥ 5	52	3514 ± 3694	4096 ± 3367	3541 ± 3274	0.729	1.49	2	63	5024 ± 5017	3517 ± 4034	4714 ± 4494	0.588	2.80	3
≥ 6	44	3676 ± 3897	4091 ± 3519	3744 ± 3412	0.764	1.24	2	62	5066 ± 5046	3511 ± 4067	4688 ± 4526	0.591	2.77	3
≥ 7	38	3340 ± 3561	4030 ± 3720	3748 ± 3484	0.815	0.91	1	62	5066 ± 5046	3511 ± 4067	4688 ± 4526	0.591	2.77	3
≥ 8	32	3382 ± 3811	4300 ± 3939	3817 ± 3733	0.827	0.84	1	61	5317 ± 5232	3522 ± 3974	4775 ± 4524	0.614	2.51	3
≥ 9	28	3750 ± 3942	4455 ± 4127	4099 ± 3891	0.831	0.81	1	61	5317 ± 5232	3522 ± 3974	4797 ± 4518	0.615	2.50	3
≥ 10	25	3998 ± 4098	4584 ± 4275	4107 ± 4011	0.847	0.72	1	61	5317 ± 5232	3522 ± 3974	4797 ± 4518	0.615	2.50	3
≥ 11	15	3855 ± 3983	4707 ± 4348	4338 ± 4584	0.875	0.57	1	59	5456 ± 5314	3524 ± 3950	4885 ± 4555	0.602	2.64	3
≥ 12	11	3270 ± 3427	4479 ± 4434	3962 ± 4576	0.839	0.77	1	53	5712 ± 5457	3601 ± 4086	5211 ± 4680	0.611	2.55	3

*ICC* Intraclass correlation coefficients, *HD* Haemodialysis. ^a^A minimum number of days needed to achieve an ICC of 0.80 calculated using the Spearman-Brown prophecy formula; ^b^Estimates from the Spearman-Brown prophecy formula should be rounded up (e.g. an estimate of at least 1.2 days should be interpreted as at least 2 days because 1 will be insufficient to achieve an ICC ≥ 0.8

**Fig. 3:**
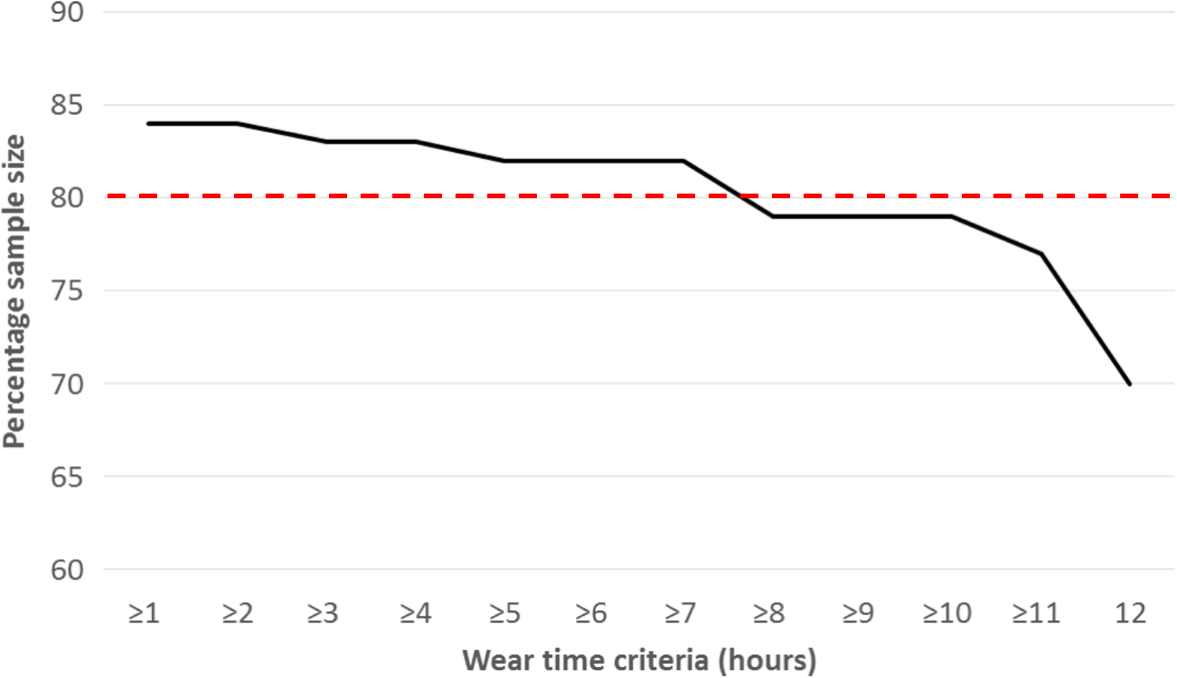
Sample attrition across a range of wear time criteria (≥ 1–12 h) when recommendations of 1 day HD and 3 days non-HD data are applied. Data are expressed as n and % of participants. The threshold for 80% sample attrition represented by the dashed line

Using criteria of at least one HD day and 3 non-HD days, the most stringent wear time criteria retaining at least 80% of the sample was ≥ 7 h (82% sample retention, Fig. 3, Additional file 4: Table S4). Therefore, at the group level, ≥ 1 HD day and ≥  3 non-HD days each with ≥ 7 h of waking wear time are recommended to obtain PA date representative of a week involving HD treatment.

## Discussion

This study is the first to provide recommendations for the measurement and processing of objective PA data in a diverse population of people receiving HD. We observed significantly reduced adherence with device wear on an HD days, partly driven by poor compliance during the HD session. Patients were less physically active on HD days compared with non-HD days. We recommend that ≥ 7 h of waking wear time on ≥1 HD day and ≥  3non-HD days is required to provide reliable PA data, whilst retaining an acceptable sample size.

The current study suggests that due to reduced variability in PA on HD days, at least 7 h of data are required on at least one HD day, and three non-HD days are required to obtain representative PA data. There is no published consensus on how many days and hours of data are required to capture representative data in an HD population, but our findings align with work in other populations living with long-term conditions. A single day of at least 11 h data was representative of PA in a cohort of inpatients living with Chronic Obstructive Pulmonary Disease, as activity restriction enforced by hospitalisation (comparable to an HD day) reduced variability in step count [38]. In the same population, in free-living conditions (akin to non-HD days in the current study), 4 days of at least 8 h of wear time have been recommended [22]. In healthy populations, a period of 2–6 days, inclusive of weekend days is required for the accurate analyses of a range of PA outcomes [39]. The specific patterns of activity between HD and non-HD days in the current study underline the need for specific measurement guidance in this population to accurately capture representative and reliable PA data.

Recommendations for capturing PA data within an HD population are summarised within Table 3. Wear time and valid day recommendations are not protocol recommendations, and study participants should wear the activity monitor for 7 days in the anticipation that not all participants will achieve this level of adherence, but may manage to wear the monitor for the minimum number of days and hours required for reliable analyses. The recommendations are of value for those who are less adherent because they outline the minimum level at which clinicians and researchers can have confidence that the data are sufficiently reliable. This is particularly relevant considering that adherence to wearing devices can be particularly low within the HD population [40]. Retaining the greatest amount of data for analysis without compromising the quality of the data and the sample size has important implications for the statistical power of a trial and the representativeness of the cohort included [22, 23].

**Table 3:** Summary of recommendations for the objective measurement of representative physical activity data in people receiving HD

Individuals should wear the monitor for a 7-day protocol	
Include data in the analysis if data satisfies the following conditions:	
• ≥ 3 non-HD days	
• ≥ 1 HD day	
• ≥ 7 h of data on either type of day	

*HD* haemodialysis

In the current study, wear-time during the HD session was low, with the device removed for 37% of the time. This may be due to the wear location of the device used. The SWA is worn on the posterior aspect of the arm, proximal to the elbow, on the non-fistula side. Consequently, participants may have been required to remove it to allow for blood pressure measurement to be taken during HD; and subsequently left it off. Other devices, such as those worn on the wrist, may not be subject to these issues. The number of wear locations and types of devices, available for monitoring physical behaviours still plagues the standardisation of physical activity measurement.

People receiving HD were inactive on all days of the week and would be classified as having a ‘sedentary lifestyle’ based on the threshold of < 5000 steps per day, regardless of the day of the week [41]. This finding is supported by previous research [3, 24]. Given the link between high levels of inactivity and poor outcomes, providing support and opportunities for people receiving HD to become more active should be an essential component of routine care. Intradialytic exercise (IDE), typically delivered during HD, is the predominant form of rehabilitation for patients [42], but the low levels of PA on non-dialysis days observed in the present study, indicate that a greater focus on increasing PA in the interdialytic period is also warranted [40, 43]. People receiving HD are less restricted within the interdialytic period and may be more able to engage in interventions covering a wider range of activities, within the context of their usual daily routine [44]. Currently, there is limited evidence that IDE directly influences habitual PA [45, 46] and relatively few trials evaluating the effects of interdialytic PA interventions for people receiving HD [46, 47]. Future trials should seek to address this gap in the literature. The use of the standardised measurement guidance for PA behaviours in HD presented here should allow for a more robust comparison of PA interventions, the impact of different regimes of dialysis (for example those undertaking twice-weekly HD, shorter sessions of HD and nocturnal HD) on PA, and will facilitate the identification of groups who may be particularly inactive.

This study is the first to report recommendations for the measurement and processing of objective PA data in the context of HD, and a main strength of our approach is the inclusion of a geographically and demographically diverse population of people receiving HD, creating robust guidance that is widely applicable. The use of step count as a marker for PA allows findings to be readily understandable and clinically meaningful. However, step count does not represent the intensity of PA, and it is unclear whether our recommendations are optimal for other PA metrics [22]. Additionally, the SWA has been shown to underestimate step count in other chronic disease populations with low walking speeds [48].

## Conclusion

PA in people receiving HD is low on all days, but particularly on days undergoing HD. When analysing objectively measured PA at group level, a wear-time of ≥ 7 h on both ≥ 1 HD day and ≥ 3 non-HD days is required to provide reliable data. Participants should also be encouraged to wear the monitor for as long as possible during the HD session. These recommendations will promote the standardised assessment of PA, enhancing data quality and thus ensuring that PA interventions can be effectively evaluated, compared and synthesised.

## Supplementary information

**Additional file 1: Table S1.** Unadjusted average daily wear time in minutes across a range of minimum wear time criteria.

**Additional file 2: Table S2.** Unadjusted average daily step count across a range of minimum wear time criteria. Data for all days, haemodialysis days (HD), weekdays (WD) and weekends (WE).

**Additional file 3: Table S3.** Average daily step count across a range of minimum wear time criteria, adjusted for wear time.

**Additional file 4: Table S4.** Sample attrition across a range of wear time criteria (≥ 1–12 h) when recommendations of 1 day HD and 3 days non-HD data are applied.

## Data Availability

The datasets used and analysed during the current study are available from the corresponding author on reasonable request.

## Abbreviations

ANCOVA: Analysis of covariance
BMI: Body mass index
CRP: C-reactive protein
ESA: Erythropoietin stimulating agent
HD: Haemodialysis
ICC: Intraclass correlation coefficient
IDE: Intradialytic exercise
NHS: National Health Service
PA: Physical activity
SWA: Sense Wear Armband
