# Supplementary material for: Standardising the measurement of physical activity in people receiving haemodialysis: considerations for research and practice
Source: BMC Nephrol. 2019 Dec 4;20:450. doi: 10.1186/s12882-019-1634-1 (PMC6894215; doi:10.1186/s12882-019-1634-1)
Supplement: Supplementary file 1 — Additional file 1: Table S1. Unadjusted average daily wear time in minutes across a range of minimum wear time criteria. [file 12882_2019_1634_MOESM1_ESM.docx]

Supplementary table 1. Unadjusted average daily wear time in minutes across a range of minimum wear time criteria. Data presented as mean (95%CI) for haemodialysis days (HD), weekdays (WD) and weekend days (WE).

| **Wear time**  **criteria** | **HD** | | **WD** | | **WE** | |
| --- | --- | --- | --- | --- | --- | --- |
|  | **N** | **Wear time (mins)** | **N** | **Wear time**  **(mins)** | **N** | **Wear time**  **(mins)** |
| **≥1 hour** | 75 | 803 (759-847)* | 71 | 951 (913-989)^a^* | 71 | 972 (934-1009)^a^ |
| **≥2 hour** | 74 | 808 (763-853) | 71 | 961 (927-996)^a^ | 71 | 972 (934-1009)^a^ |
| **≥3 hour** | 74 | 815 (771-859) | 71 | 968 (935-1001)^a^ | 70 | 972 (934-1009)^a^ |
| **≥4 hour** | 72 | 820 (775-865) | 71 | 968 (935-1001)^a^ | 70 | 972 (934-1009)^a^ |
| **≥5 hour** | 70 | 833 (790-876) | 71 | 968 (935-1001)^a^ | 70 | 972 (934-1009)^a^ |
| **≥6 hour** | 70 | 852 (811-894) | 71 | 978 (948-1008)^a^ | 70 | 972 (934-1009)^a^ |
| **≥7 hour** | 70 | 870 (830-909) | 71 | 978 (948-1008)^a^ | 70 | 972 (934-1009)^a^ |
| **≥8 hour** | 68 | 887 (850-925) | 70 | 982 (951-1012)^a^ | 69 | 977 (942-1012)^a^ |
| **≥9 hour** | 67 | 906 (869-943) | 70 | 982 (951-1012)^a^ | 68 | 977 (942-1012)^a^ |
| **≥10 hour** | 67 | 926 (893-960) | 69 | 982 (951-1012)^a^ | 67 | 977 (942-1012) |
| **≥11 hour** | 66 | 950 (919-982) | 68 | 987 (957-1017) | 66 | 978 (944-1013) |
| **12 hour** | 63 | 964 (931-997) | 68 | 994 (966-1022) | 63 | 976 (946-1013) |

^a^ p<0.05 difference compared with HD day for that wear time criteria, * p<0.05 difference between ≥1 hour wear time criteria compared with ≥12 hour criteria.
