# Supplementary material for: Standardising the measurement of physical activity in people receiving haemodialysis: considerations for research and practice
Source: BMC Nephrol. 2019 Dec 4;20:450. doi: 10.1186/s12882-019-1634-1 (PMC6894215; doi:10.1186/s12882-019-1634-1)
Supplement: Supplementary file 2 — Additional file 2: Table S2. Unadjusted average daily step count across a range of minimum wear time criteria. Data for all days, haemodialysis days (HD), weekdays (WD) and weekends (WE). [file 12882_2019_1634_MOESM2_ESM.docx]

Supplementary table 2. Unadjusted average daily step count across a range of minimum wear time criteria. Data for all days, haemodialysis days (HD), weekdays (WD) and weekends (WE).

|  | **N** | **HD** | **N** | **WD** | **N** | **WE** |
| --- | --- | --- | --- | --- | --- | --- |
| **Wear time criteria** |  | **Daily step count** |  | **Daily step count** |  | **Daily step count** |
| **≥1 hour** | 75 | 3402(2665-4140) | 71 | 4914 (3940-5887)^a^ | 71 | 4633 (3558-5707)^a^ |
| **≥2 hour** | 74 | 3411 (2675-4147) | 71 | 5045 (4018-6071)^a^ | 71 | 4633 (3558-5707)^a^ |
| **≥3 hour** | 74 | 3440 (2704-4176) | 71 | 5083 (4060-6107)^a^ | 70 | 4633 (3558-5707)^a^ |
| **≥4 hour** | 74 | 3474 (2731-4216) | 71 | 5083 (4060-6107)^a^ | 70 | 4633 (3558-5707)^a^ |
| **≥5 hour** | 72 | 3497 (2759-4236) | 71 | 5083 (4060-6107)^a^ | 70 | 4633 (3558-5707)^a^ |
| **≥6 hour** | 70 | 3567 (2823-4312) | 71 | 5090 (4064-6116)^a^ | 70 | 4633 (3558-5707)^a^ |
| **≥7 hour** | 69 | 3666 (2893-4439) | 71 | 5090 (4064-6116)^a^ | 70 | 4633 (3558-5707)^a^ |
| **≥8 hour** | 68 | 3676 (2906-4447) | 70 | 5142 (4093-6191)^a^ | 69 | 4695 (3621-5769)^a^ |
| **≥9 hour** | 67 | 3759 (2987-4532) | 70 | 5142 (4093-6191)^a^ | 68 | 4695 (3621-5769)^a^ |
| **≥10 hour** | 67 | 3955 (3120-4791) | 69 | 5142 (4093-6191)^a^ | 67 | 4695 (3621-5769) |
| **≥11 hour** | 66 | 4083 (3206-4959) | 68 | 5170 (4121-6219)^a^ | 66 | 4701 (3629-5773) |
| **12 hour** | 63 | 4094 (3219-4968)* | 66 | 5175 (4126-6225)^a^ | 63 | 4711 (3641-5781) |

^a^ p<0.05 compared with HD day for that wear time criteria, * p<0.05 between ≥1 hour wear time criteria compared with ≥12 hour criteria.
