# Supplementary material for: Standardising the measurement of physical activity in people receiving haemodialysis: considerations for research and practice
Source: BMC Nephrol. 2019 Dec 4;20:450. doi: 10.1186/s12882-019-1634-1 (PMC6894215; doi:10.1186/s12882-019-1634-1)
Supplement: Supplementary file 3 — Additional file 3: Table S3. Average daily step count across a range of minimum wear time criteria, adjusted for wear time. [file 12882_2019_1634_MOESM3_ESM.docx]

Supplementary table 3. Average daily step count across a range of minimum wear time criteria, adjusted for wear time. Data presented as mean (95%CI) for all days, haemodialysis days (HD), weekdays (WD) and weekends (WE).

| **Wear time criteria** | **N** | **HD Daily step count** | **N** | **WD Daily step count** | **N** | **WE Daily step count** |
| --- | --- | --- | --- | --- | --- | --- |
| **≥1 hour** | 75 | 3318  (2610-4026)* | 71 | 4859  (3882-5827)^a^ | 71 | 4607  (3586-5629)^a^ |
| **≥2 hour** | 74 | 3326  (2622-4030) | 71 | 4984  (3959-6009)^a^ | 71 | 4607  (3586-5629)^a^ |
| **≥3 hour** | 74 | 3354  (2649-4059) | 71 | 5022  (3999-6045)^a^ | 70 | 4607  (3585-5629)^a^ |
| **≥4 hour** | 74 | 3387  (2677-4097) | 71 | 5022  (3999-6045)^a^ | 70 | 4607  (3585-5629)^a^ |
| **≥5 hour** | 72 | 3487  (2766-4209) | 71 | 5029  (3990-6067)^a^ | 70 | 4625  (3587-5663)^a^ |
| **≥6 hour** | 70 | 3566  (2838-4293) | 71 | 5035  (3995-6076)^a^ | 70 | 4625  (3587-5663)^a^ |
| **≥7 hour** | 69 | 3634  (2873-4396) | 71 | 5045  (3989-6100)^a^ | 70 | 4595  (3546-5645)^a^ |
| **≥8 hour** | 68 | 3676  (2908-4445) | 70 | 5161  (4071-6250)^a^ | 69 | 4710  (3649-5771)^a^ |
| **≥9 hour** | 67 | 3759  (2987-4532) | 70 | 5161  (4071-6350)^a^ | 68 | 4710  (3649-5771)^a^ |
| **≥10 hour** | 67 | 3910  (3080-4739) | 69 | 5161  (4073-6248)^a^ | 67 | 4710  (3652-5768) |
| **≥11 hour** | 66 | 4116  (3223-5008) | 68 | 5289  (4170-6408)^a^ | 66 | 4791  (3705-5877) |
| **12 hour** | 63 | 4113  (3166-5060) | 66 | 5238  (4045-6431)^a^ | 63 | 4870  (3708-6031) |

^a^ p<0.05 difference to HD day, *p<0.05 difference compared with ≥12 hour criteria.
