# Supplementary material for: Standardising the measurement of physical activity in people receiving haemodialysis: considerations for research and practice
Source: BMC Nephrol. 2019 Dec 4;20:450. doi: 10.1186/s12882-019-1634-1 (PMC6894215; doi:10.1186/s12882-019-1634-1)
Supplement: Supplementary file 4 — Additional file 4: Table S4. Sample attrition across a range of wear time criteria (≥ 1–12 h) when recommendations of 1 day HD and 3 days non-HD data are applied. [file 12882_2019_1634_MOESM4_ESM.docx]

Supplementary table 4. Sample attrition across a range of wear time criteria (≥ 1-12 hours) when recommendations of 1 day HD and 3 days non-HD data are applied. Data are expressed as n and % of participants.

|  | **≥1 hour** | **≥2 hour** | **≥3 hour** | **≥4 hour** | **≥5 hour** | **≥6 hour** | **≥7 hour** | **≥8 hour** | **≥9 hour** | **≥10 hour** | **≥11 hour** | **12 hour** |
| --- | --- | --- | --- | --- | --- | --- | --- | --- | --- | --- | --- | --- |
| **n** | 65 | 65 | 64 | 64 | 63 | 63 | 63 | 61 | 61 | 61 | 59 | 54 |
| **%** | 84 | 84 | 83 | 83 | 82 | 82 | 82 | 79 | 79 | 79 | 77 | 70 |
